# Supplementary material for: Purkinje cell outputs selectively inhibit a subset of unipolar brush cells in the input layer of the cerebellar cortex
Source: eLife. 2021 Aug 9;10:e68802. doi: 10.7554/eLife.68802 (PMC8352585; doi:10.7554/eLife.68802)
Supplement: Transparent reporting form [file elife-68802-transrepform.docx]

***eLife’s* transparent reporting form**

We encourage authors to provide detailed information *within their submission* to facilitate the interpretation and replication of experiments. Authors can upload supporting documentation to indicate the use of appropriate reporting guidelines for health-related research (see [EQUATOR Network](http://www.equator-network.org/%20)), life science research (see the [BioSharing Information Resource](https://biosharing.org/)), or the [ARRIVE guidelines](http://www.plosbiology.org/article/info:doi/10.1371/journal.pbio.1000412) for reporting work involving animal research. Where applicable, authors should refer to any relevant reporting standards documents in this form.

If you have any questions, please consult our Journal Policies and/or contact us: [editorial@elifesciences.org](mailto:editorial@elifesciences.org).

**Sample-size estimation**

- You should state whether an appropriate sample size was computed when the study was being designed
- You should state the statistical method of sample size computation and any required assumptions
- If no explicit power analysis was used, you should describe how you decided what sample (replicate) size (number) to use

Please outline where this information can be found within the submission (e.g., sections or figure legends), or explain why this information doesn’t apply to your submission:

No explicit sample size calculation was made. For correlating glutamatergic synapse phenotype with presence of inhibitory feedback we sampled large numbers (n=41 and 60) using two independent measurements glutamate or DHPG. For paired recordings, only two cells were obtained as the success rate was extremely low across several weeks of experiments (2/~400). However, this is sufficient to qualitatively confirm the existence of this mono-synaptic feedback.

**Replicates**

- You should report how often each experiment was performed
- You should include a definition of biological versus technical replication
- The data obtained should be provided and sufficient information should be provided to indicate the number of independent biological and/or technical replicates
- If you encountered any outliers, you should describe how these were handled
- Criteria for exclusion/inclusion of data should be clearly stated
- High-throughput sequence data should be uploaded before submission, with a private link for reviewers provided (these are available from both GEO and ArrayExpress)

Please outline where this information can be found within the submission (e.g., sections or figure legends), or explain why this information doesn’t apply to your submission:

All cells are included. In the optogenetic experiment the slices are pre-screened for health by measuring optical responses in Purkine cells as outlined in the method section.

**Statistical reporting**

- Statistical analysis methods should be described and justified
- Raw data should be presented in figures whenever informative to do so (typically when N per group is less than 10)
- For each experiment, you should identify the statistical tests used, exact values of N, definitions of center, methods of multiple test correction, and dispersion and precision measures (e.g., mean, median, SD, SEM, confidence intervals; and, for the major substantive results, a measure of effect size (e.g., Pearson's r, Cohen's d)
- Report exact p-values wherever possible alongside the summary statistics and 95% confidence intervals. These should be reported for all key questions and not only when the p-value is less than 0.05.

Please outline where this information can be found within the submission (e.g., sections or figure legends), or explain why this information doesn’t apply to your submission:

For anatomical results, the lobule-wise distribution is directly visualized by bouton and synapse density. For all physiology data, individual cells are displayed in all cases. Summaries used mean+/-sem as indicated in the main text and method. No statistical tests are used.

(For large datasets, or papers with a very large number of statistical tests, you may upload a single table file with tests, Ns, etc., with reference to sections in the manuscript.)

**Group allocation**

- Indicate how samples were allocated into experimental groups (in the case of clinical studies, please specify allocation to treatment method); if randomization was used, please also state if restricted randomization was applied
- Indicate if masking was used during group allocation, data collection and/or data analysis

Please outline where this information can be found within the submission (e.g., sections or figure legends), or explain why this information doesn’t apply to your submission:

Most data came from wild type mice. In optogenetics experiments, pcp2-crexchr2 animals were used exclusively and control was performed in within the same cell in a method identical to (Guo et al., 2016).

Molecular data for cerebellar UBCs can be visualized through (<https://singlecell.broadinstitute.org/single_cell/study/SCP795/>). Raw and processed data that support the findings of this study have been deposited in GEO under accession number GSE165371 and in at the Neuroscience Multi-omics (NeMO) Archive (<https://nemoarchive.org/>). Figure2-figure supplementary 1 (mglur1) subpanel is from (Guo et al., 2020)

**Additional data files (“source data”)**

- We encourage you to upload relevant additional data files, such as numerical data that are represented as a graph in a figure, or as a summary table
- Where provided, these should be in the most useful format, and they can be uploaded as “Source data” files linked to a main figure or table
- Include model definition files including the full list of parameters used
- Include code used for data analysis (e.g., R, MatLab)
- Avoid stating that data files are “available upon request”

Please indicate the figures or tables for which source data files have been provided:

Additional physiology figure source data will be made available on <https://github.com/chongguo>.
